# Extra-Uterine Pregnancy

**Published:** 1876-04

**Authors:** 


*Extra-Uterine Pregnancy: its Causes, Species, Pathological Anatomy, Prognosis and Treatment.* By John S. Parry, M. D. Philadelphia: Henry C. Lea. 1876. Buffalo: T. Butler & Son.

This work is based on an analysis of five hundred cases of extra-uterine pregnancy, collected from the reports of various authors.

The author says that the cause in many instances cannot be determined. Among the causes which have been recognized as producing a misplaced pregnancy he places the results of previous pelvic disease: constriction and displacement of the uterine appendages. Inaptitude to conception, hernia of the internal genital organs, displacements and tumors of the uterus, operations on the uterus, moral and emotional causes, twin conceptions, diseases and deranged action of the fallopian tubes, age and the number of the pregnancy are also cited as causes of extra-uterine pregnancy. In reference to operations upon the uterus, the author cites two somewhat remarkable cases. One which was observed by M. Kœberle is as follows: This surgeon removed all the body and part of the neck of the uterus on account of a fibroïd tumor. The appendages of the uterus were left. The patient recovered with a fistula through the cicatrix of the neck by which she became pregnant. She went to term and died undelivered.

Of the varieties of extra-uterine pregancy, the author recognizes but three: tubal, ovarian and ventral or abdominal. Each of these he divides into several classes.

In the estalishment of the connection between the ovum and its abnormal location, but one force is concerned. This is a vital adhesion between the villi of the chorion and the tissue with which they come in contact, aided by the presence of more or less plastic material. It differs from the same process in the uterus in the absence of the decidua, the subovular portion of which—called the serotina—plays an important part in establishing a connection between the ovular and maternal tissues by means of the active proliferation of its cells.

In regard to the uterus, it seems that it undergoes changes nearly similar to those which are observed in the normal gestations. The author lays down the following propositions: 1. In all varieties of extra-uterine pregnancy a decidua forms in the uterine cavity, as in normal gestation, but none surrounds the ovum. 2. The decidua is rarely retained until the completion of gestation, and thrown off during false labor. More frequently, if the patient goes to term, it is discharged during the early periods of pregnancy in small fragments, and without producing pain; or else it is expelled *en masse* with symptoms of miscarriage. 3. The absence of a uterine decidua when death has occurred from rupture of the cyst, even in the early stages of pregnancy, is not proof that the membrane has not been formed, but simply that it has been expelled before the death of the patient.

The symytoms of extra-uterine pregnancy are all carefully studied, and are divided into those evinced at three periods. 1. Those at the beginning of the pregnamcy, when the fœtal heart cannot be heard. 2. After the period when the fœtal heart is audible, and until after the close of the spurious labor, or at term. 3. After the temination of the false labor, or the death of the fœtus.

In the termination of a case of this character there are three conditions which the author describes: Rupture of the cyst, changes which follow retention of the fœtus for a long period, and discharge of the child through the bowel, bladder, vagina or abdominal wall. Of the five hundred cases which the author has collected, three hundred and thirty-six proved fatal—a death rate of over sixty-seven per cent.

The various procedures which have been adopted in these cases are considered in detail. The author believes it to be the duty of the attendant, in cases of this character, where there is unmistakable evidence of rupture of the cyst, to open the abdominal cavity and remove the contents of the ruptured cyst, thus attempting to save the patient.

This work is one of extreme value, and shows evidence of a large amount of patient work. We are only sorry the author could not have been spared to reap some of the results of his labors.

